# Two Decades of Land Subsidence in Tianjin, China, Measured with Multi-Temporal InSAR Observations

**DOI:** 10.3390/s26041203

**Published:** 2026-02-12

**Authors:** Haolin Zhao, Hongyue Zhou, Dashan Zhou, Chaoying Zhao

**Affiliations:** 1 School of Geological Engineering and Geomatics, Chang’an University, Xi’an 710054, China; 2022126049@chd.edu.cn; 2 Tianjin Surveying and Mapping Institute Co., Ltd., Tianjin 300381, China; ZhouHY0516@126.com (H.Z.); zhoudashan@whu.edu.cn (D.Z.); 3 Tianjin Key Laboratory of Spatio-Temporal Information Engineering and Technology, Tianjin 300381, China; 4 Key Laboratory of Western China’s Mineral Resources and Geological Engineering, Ministry of Education, Xi’an 710054, China

**Keywords:** SBAS-InSAR, logistic model, long time series, land subsidence, Tianjin

## Abstract

Land subsidence poses a persistent challenge to Tianjin, a major coastal city in China, with implications for urban infrastructure and sustainable development. This study examines the spatiotemporal evolution of ground subsidence in Tianjin from 2003 to 2024 using multi-source SAR observations from Envisat ASAR (C-band), ALOS PALSAR (L-band), and Sentinel-1 (C-band). Surface deformation was derived using SBAS-InSAR with atmospheric phase correction. Due to limitations in data availability, SAR observations are temporally discontinuous; therefore, the long-term subsidence evolution was reconstructed by integrating multi-sensor deformation rates through a model-based time-series fitting approach. The results show pronounced subsidence during 2003–2010 in inland districts such as Wuqing, Beichen, Jinnan, and Jinghai, with maximum rates exceeding 50 mm/yr. After 2017, regional subsidence rates generally declined, while localized deformation became increasingly concentrated in coastal reclamation areas of the Binhai New Area, particularly around Dongjiang Port and Fuzhuang. Spatial and temporal patterns of subsidence exhibit clear correspondence with changes in groundwater use intensity and phases of urban construction and land reclamation. These observations suggest a transition in dominant subsidence controls over time. The results provide a long-term observational perspective on subsidence evolution in Tianjin and offer a geospatial basis for land-use planning and infrastructure risk assessment in coastal cities.

## 1. Introduction

Ground subsidence is a widespread geological hazard that commonly occurs in regions characterized by intensive groundwater extraction, unconsolidated sedimentary formations, and rapid urbanization. This phenomenon poses a serious threat and consequently constrains sustainable socio-economic development in affected areas [1]. Ground subsidence is typically a slow and progressive process with a broad spatial extent, sometimes covering tens to hundreds of kilometers, and results surface elevation changes [2]. Although natural compaction contributes to subsidence, human activities can significantly accelerate this process [3]. Uneven ground deformation may further induce secondary hazards such as ground fissures and structural damage to buildings and infrastructure. Previous studies have reported severe land subsidence in many regions worldwide, including Japan, the US, Italy and China, with an increasing number of related hazards attributed to anthropogenic activities such as groundwater and underground resource extraction, subsurface space development, and large-scale surface construction [3,4,5,6,7].

In China, ground subsidence is particularly pronounced in rapidly urbanizing regions, including the Beijing-Tianjin-Hebei area, the Yangtze River Delta, and the North China Plain [2]. Tianjin, one of China’s major coastal economic centers, is located in a typical low-lying plain composed of thick unconsolidated sediments. Since the mid-20th century, the city has experienced the development of multiple subsidence centers due to long-term groundwater over-extraction combined with unfavorable geological conditions. In recent decades, rapid urban expansion and intensive infrastructure construction have further exacerbated subsidence processes, leading to substantial economic losses and increasing risks to transportation networks, pipelines, subways, and buildings [8,9,10].

In recent years, Synthetic Aperture Radar Interferometry (InSAR) has become an essential technique for monitoring ground subsidence owing to its high spatial resolution, wide-area coverage, and centimeter-level deformation measurement capability. With the continuous advancement of satellite missions and processing algorithms, time-series InSAR techniques such as Persistent Scatterers InSAR (PS-InSAR) [11] and Small Baseline Subset (SBAS-InSAR) [12], have been widely applied to extract deformation signals from high-coherence targets, including buildings and roads. However, conventional methods still face limitations in low-coherence areas, such as urban fringes and coastal zones, where monitoring points are sparse and deformation fields tend to be spatially discontinuous. To address this, the Intermittent Small Baseline Subset (ISBAS) method has been proposed and increasingly applied in low-coherence environments, significantly improving the spatial continuity of subsidence monitoring results [13,14]. Moreover, the continuous launch of SAR satellites has expanded both the temporal and spatial coverage of InSAR observations, making the joint processing of multi-source SAR data an effective approach for enhancing spatial coverage and improving the consistency of deformation time-series [15,16,17].

Despite these advances, most existing studies on ground subsidence in Tianjin have focused on relatively short time periods and have primarily relied on single-sensor SAR datasets. Few studies have attempted to integrate multi-sensor and multi-band InSAR observations, and no comprehensive investigation has reconstructed ground deformation patterns in Tianjin by linking multi-source SAR data over a long temporal span from 2003 to 2024. Consequently, the long-term evolution of subsidence patterns and their associated driving factors remain insufficiently understood.

To address these limitations, this study aims to reconstruct the spatiotemporal evolution of ground subsidence in Tianjin over the period from 2003 to 2024 using multi-source SAR data. Rather than continuous monitoring throughout the entire period, this work integrates deformation information derived from different sensors to characterize the long-term subsidence trends across two decades. By combining multi-temporal InSAR observations, this study provides a comprehensive assessment of historical and recent subsidence patterns and analyzes the key natural and anthropogenic factors influencing ground subsidence in Tianjin.

## 2. Study Area

### 2.1. Study Area Background

**Figure 1:**
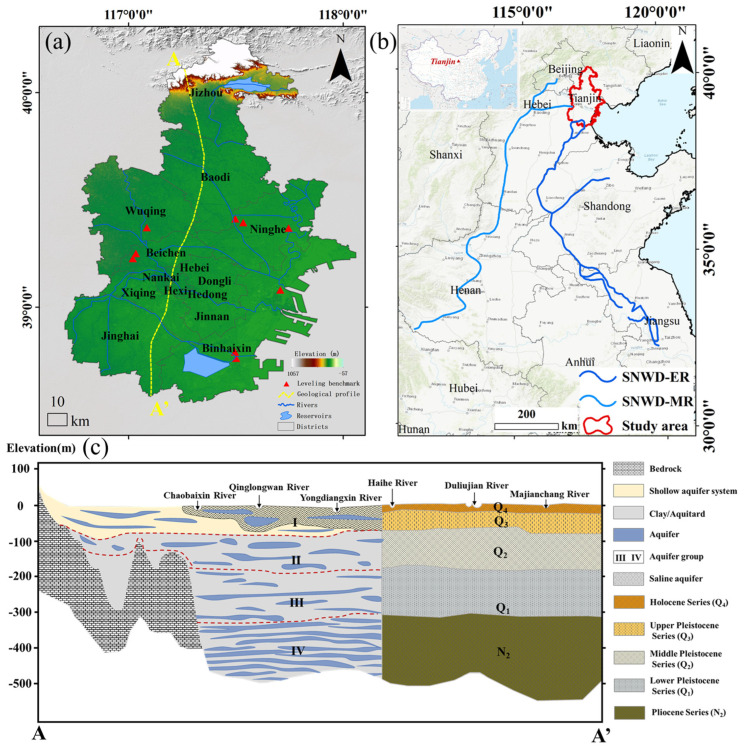
Overview of study area. (**a**) The geographical location and coverage of Tianjin. (**b**) The relative position of the SNWD’s middle and eastern routes to Tianjin. (**c**) A typical Quaternary geological section in Tianjin cross section A-A’ in (**a**) [8,9,10].

Tianjin is located in the eastern part of the North China Plain, on the western coast of the Bohai Bay. It is situated between 116°43′ to 118°04′ E longitude and 38°34′ to 40°15′ N latitude, covering an area of approximately 12,000 square kilometers. The region is characterized by low-lying terrain, with an elevation gradually decreasing from the northwest to the southeast. Most of the surface elevation is between 5 and 10 m, and it is a typical coastal alluvial and marine sedimentary plain (Figure 1a). Tianjin has 16 administrative districts, with a densely populated central urban area surrounded by agricultural and industrial zones. The city has seen rapid urbanization and significant infrastructure development along rivers and coasts.

Geologically, the underground structure of Tianjin is primarily composed of thick Neogene to Quaternary sediment layers, with sediment thickness generally ranging from 300 to 600 m. The critical engineering layers lie within the upper 50 m [18]. The Quaternary strata are characterized by multiple sand aquifers interspersed with fine silty and clayey aquicludes, forming a typical confined aquifer system [19]. Under the condition of excessive groundwater extraction, this structure is highly susceptible to compression subsidence, especially when the water levels in the aquifers decline over extended periods, leading to non-elastic subsidence.

Hydrologically, Tianjin’s groundwater is primarily from confined aquifers, distributed between the Ι and ΙV aquifer layers [19]. In recent years, due to the increasing demand for industrial and urban water, the groundwater level has significantly dropped. Since 2014, the South-to-North Water Diversion Project (SNWD) has provided a stable water source for Tianjin’s industrial and domestic water supply, alleviating the pressure on groundwater extraction [20] (Figure 1b). Additionally, restrictions on groundwater extraction in certain areas, such as Wuqing District and Binhai New Area, along with ecological replenishment measures, have contributed to the recovery of groundwater levels.

### 2.2. Ground Subsidence History and Evolution in Tianjin

Tianjin was one of the first cities in China to observe ground subsidence. As early as the 1920s, subsidence was noted in some areas due to the development of confined groundwater. The subsidence rate in the city ranged from 7.1 to 12 mm/yr [21,22]. Following the advancement of urban infrastructure and the continued development of deep groundwater resources, ground subsidence became more pronounced. From the 1950s onward, excessive groundwater extraction caused widespread subsidence in the Tianjin Plain, forming five major subsidence centers in the urban area, Tanggu, Hangu, Dagang, and the Haihe River downstream industrial areas. Between 1959 and 1983, the cumulative subsidence in Tianjin city reached 2.27 m, in Tanggu 2.13 m, and in Hangu 1.89 m [23,24].

**Figure 2:**
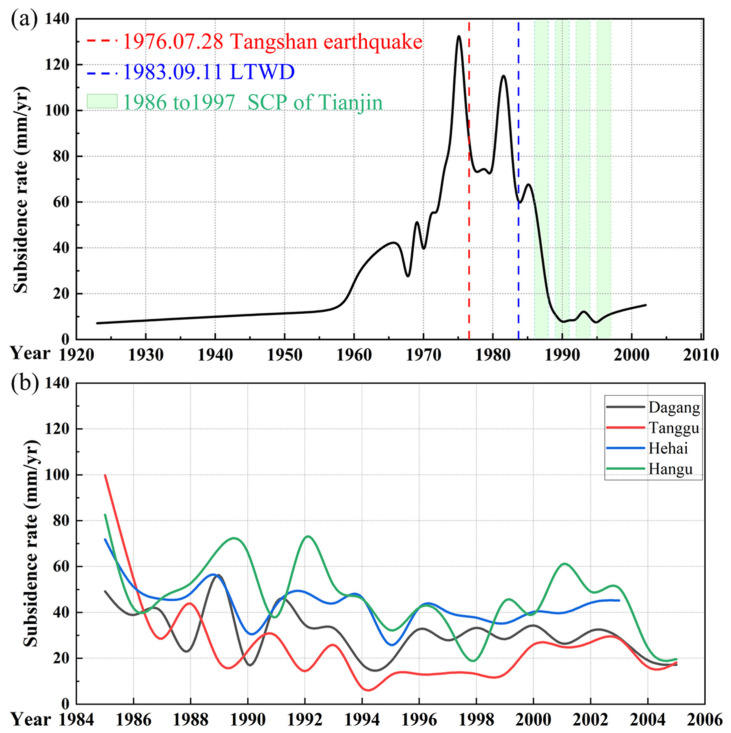
The historical subsidence of important cities statistics in Tianjin. (**a**) The rate of subsidence in the center of Tinajin from 1923 to 2002. (**b**) The changes in the subsidence funnels of Dagang, Tanggu, Hangu, and the Haihe River downstream industrial areas from 1985 to 2005 [22,24,25].

Moreover, since the late Neogene period, the earth’s crust in the Tianjin Plain has been in a subsiding state, with relative tectonic activity between different structural units. A significant example of such activity occurred during the 1976 Tangshan earthquake (The red dotted line in Figure 2a), where the area around Fuzhuang in Hangu underwent vertical deformation of up to 3 m, and the embankment along the Caijiabu to Dashantang region experienced subsidence of more than 1 m [23].

Since the 1980s, groundwater extraction in Tianjin increased rapidly, exacerbating the ground subsidence issue. However, in 1983, the Luanhe-Tianjin Water Diversion Project (LTWD) (The blue dotted line in Figure 2a) was implemented, which alleviated the water scarcity issue in Tianjin and partially reduced the subsidence trend. In 1986, Tianjin initiated its first settlement control plan (SCP), which aimed to reduce subsidence by limiting groundwater extraction and implementing recharge measures. The city also established a hydrological and geological observation network and became the first in China to conduct subsidence cause analysis and control pilot projects, thus accumulating valuable experience in subsidence research. Between 1986 and 1997, Tianjin carried out four phases of a three-year subsidence control program (Figure 2a The green rectangle covers the time period), which significantly improved the subsidence situation and laid the foundation for subsequent control efforts [24]. Although the settlement control plan achieved some success, the continued decline of groundwater levels in many areas still led to subsidence, particularly in regions that heavily relied on groundwater resources.

After 2000, with the ongoing over-extraction of groundwater, Tianjin’s subsidence rate was among the highest in China, with some areas experiencing subsidence rates exceeding 50 mm/yr. According to the National Ground Subsidence Monitoring and Early Warning System, the cumulative subsidence in Tianjin between 2000 and 2010 was among the highest in the country, with a subsidence area of over 4000 square kilometers. After the South-to-North Water Diversion Project was officially implemented in 2014, the pressure on groundwater extraction in Tianjin was greatly alleviated, leading to a noticeable rebound in groundwater levels [20]. The transition from groundwater supply to external water resources effectively curbed the continued decline of groundwater levels and alleviated the subsidence trend [16,26]. Moreover, ecological replenishment projects have also helped mitigate ground subsidence, with some regions even experiencing uplift. However, due to the continued compression effect in the strata, subsidence has not fully stopped, especially in areas with thick sediment layers and complex aquifer systems, where some regions still exhibit the lagging subsidence.

Overall, Tianjin’s ground subsidence issue results from a combination of natural and human-induced factors, including groundwater extraction, geological structures, and policy changes. Since 1923, with the implementation of significant engineering projects and policies in different historical periods, Tianjin’s ground subsidence evolution has shown distinct stages, and the subsidence issue has gradually improved. However, there are still complex challenges ahead.

## 3. Data and Methodology

**Figure 3:**
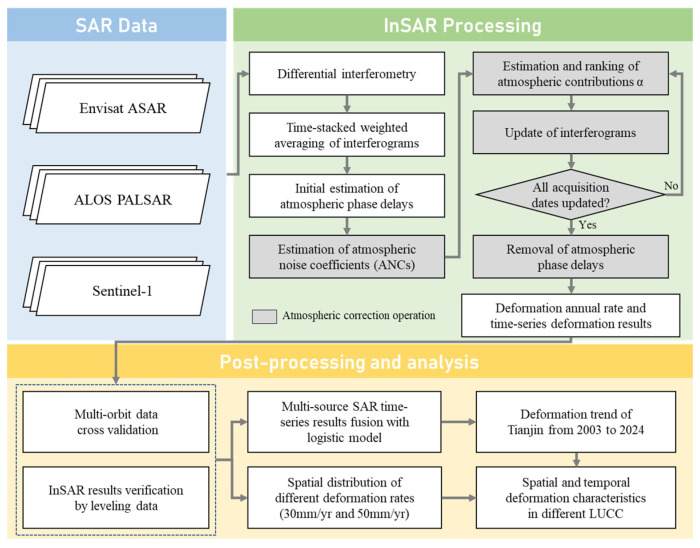
The workflow of two decades land deformation research in Tianjin.

This section describes the SAR datasets used in this study and the corresponding data processing and analysis methods. An overview of the entire processing workflow is illustrated in Figure 3. The “SAR Data” component corresponds to Section 3.1, which introduces the characteristics of the multi-source SAR datasets. The “InSAR Processing” component covers the InSAR processing procedures described in Section 3.2 and Section 3.3. Among these, atmospheric phase correction is a critical step in InSAR processing; therefore, the adopted atmospheric correction strategy is described in detail in Section 3.3. The “Post-processing and analysis” component corresponds to Section 3.4, which presents the multi-source data fusion model.

All SAR data processing and InSAR time-series analyses were performed using the GAMMA (Version 2020) [27] software. MATLAB (R2023b) [28] was used for the extraction and visualization of deformation time series at representative points, while Python (Version 3.8) [29] was employed to construct the Logistic S-curve model and to integrate deformation time-series results derived from different SAR datasets.

### 3.1. Data

To analyze ground subsidence in Tianjin from 2003 to 2024, this study utilized multi-source Synthetic Aperture Radar (SAR) data acquired from three satellite missions: Envisat ASAR (C-band) by European Space Agency (ESA), ALOS PALSAR (L-band) by Japan Aerospace Exploration Agency (JAXA), and Sentinel-1 (S1) (C-band) by ESA. These datasets cover different observation periods and sensor characteristics, providing complementary information for investigating the long-term spatiotemporal evolution of land subsidence in Tianjin (Table 1).

**Figure 4:**
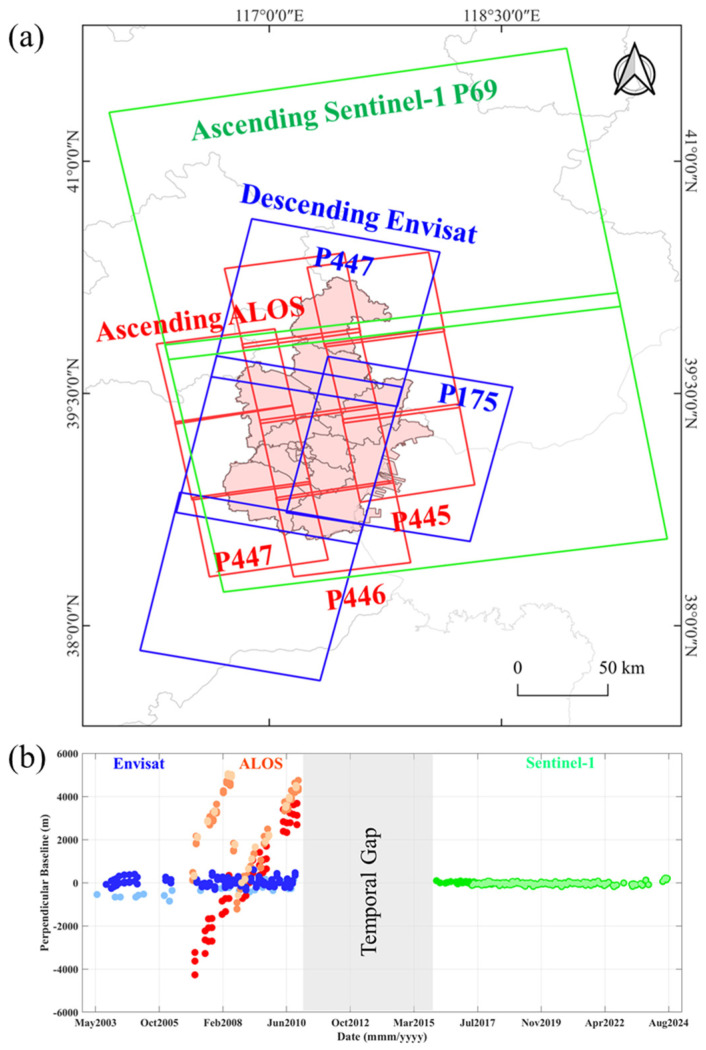
SAR dataset temporal and spatial coverage situation. (**a**) SAR data coverage. (**b**) SAR data temporal and spatial distribution.

Specifically, the Envisat ASAR data span from 2003 to 2010, the ALOS PALSAR data cover the period from 2007 to 2010, and the Sentinel-1 data extend from 2016 to 2024. Due to the availability of free SAR archives, no SAR observations were available for the period from 2011 to 2015, resulting in a temporal gap in the long-term deformation record. The spatial coverage and temporal distribution of the SAR datasets are illustrated in Figure 4a. For Sentinel-1, two adjacent frames were acquired with a temporal offset exceeding one year; therefore, only their overlapping acquisition periods were used to ensure temporal consistency in the deformation analysis. It should be noted that although the Envisat ASAR dataset covers the period from 2003 to 2010, different sub-periods are used for different analytical purposes in this study. In particular, the Envisat results for 2003–2007 are presented to illustrate the stage spatial characteristics of land subsidence, while the Envisat data from 2007 to 2010 are specifically used for comparison with ALOS PALSAR during their overlapping observation period.

In addition to the SAR datasets, ground-based leveling measurements collected at nine stations in Tianjin between 2015 and 2023 were obtained from the Tianjin Surveying and Mapping Institute Figure 1a. These in situ observations were used to validate and cross-check the InSAR-derived deformation results, as described in Section 4.2. Furthermore, historical high-resolution optical imagery from Google Earth was employed as auxiliary information to support the qualitative interpretation of land-use changes and urban construction processes discussed in Section 5.1. The deformation rates derived from Sentinel-1 and the multi-source SAR time-series results obtained through the logistic S-curve fusion model represent derived products based on the InSAR processing workflow described in Section 3.2, Section 3.3 and Section 3.4, and form the basis for the spatiotemporal analyses presented in Section 4 and Section 5.

### 3.2. InSAR Data Processing

For the measurement of surface deformation, we applied the SBAS-InSAR technique to process the multi-temporal SAR data. For each sensor, SAR images acquired along the same orbit were selected to construct interferometric networks under temporal and perpendicular baseline constraints. Specifically, for each SAR scene, interferometric pairs were formed between that scene and the subsequent five temporally adjacent scenes, ensuring adequate interferogram density in temporally sparse periods while limiting temporal decorrelation. Each interferometric contains phase contributions from multiple sources, including surface deformation, atmospheric delays, orbital errors, topographic phase, and noise. The total interferometric phase ψ can be expressed as:

(1) $$\psi =\psi_{t_{B}}-\psi_{t_{A}}\approx \varphi_{def}+\varphi_{ref}+\varphi_{top}+\varphi_{atm}+\varphi_{noi}$$

where $t_{A}$ and $t_{B}$ denote the acquisition times of the two SAR images forming the interferogram, $\varphi_{def}$ represents the deformation phase, $\varphi_{ref}$ is the reference ellipsoid phase, $\varphi_{top}$ is the topographic phase, $\varphi_{atm}$ denotes the atmospheric phase delay, $\varphi_{noi}$ accounts for random noise.

To improve phase quality and spatial consistency, multi-look processing was applied to the interferograms. The multi-look ratios were set to 2:10 (range:azimuth) for Envisat ASAR, 2:8 for ALOS PALSAR, and 10:2 for Sentinel-1, resulting in an approximate ground resolution of 50 m × 50 m. For the ALOS PALSAR dataset, both FBS and FBD acquisition modes were used; prior to interferometric processing, the FBD data were resampled to match the spatial resolution of the FBS mode. A coherence threshold of 0.3 was adopted to retain distributed scatterer (DS) pixels while preserving sufficient spatial coverage. Water bodies were masked prior to phase unwrapping to reduce unwrapping errors associated with low-coherence areas. A 30 m-resolution SRTM DEM was used to estimate the topographic phase and for geocoding. Adaptive filtering was applied to the interferograms to suppress phase noise, followed by phase unwrapping using the minimum cost flow (MCF) algorithm [30]. The unwrapped interferograms were then screened to remove outliers and poorly connected interferograms before subsequent processing.

After phase unwrapping, atmospheric phase delays were estimated and corrected, as described in Section 3.3. The deformation velocity and displacement time series were then calculated through SBAS inversion. However, for datasets with relatively sparse interferometric networks—particularly Envisat ASAR in certain frames—the standard SBAS approach may lead to incomplete temporal sampling. To address this issue, the ISBAS method was employed to relax coherence requirements and incorporate intermittently coherent DS pixels, thereby improving temporal continuity and spatial coverage of the deformation time series [31].

Assuming one generic point $\left(i,j\right)$ for the kth unwrapped interferogram, the following equation is established to estimate the linear velocity $V_{ij}$ and height error ${\delta h}_{ij}$:

(2) $$\Phi_{ij}=\frac{4\pi }{\lambda }\left({\delta t}_{k}V_{ij}+\frac{B_{⊥}^{ijk}}{R_{ij}{sin\theta }_{ij}}{\delta h}_{ij}\right)$$

where $\Phi_{ij}$ is the phase of the interferogram, λ is the radar wavelength, ${\delta t}_{k}$ is the time difference, $B_{⊥}^{ijk}$ is the perpendicular baseline, $R_{ij}$ is the slant range of the target, and $\theta_{ij}$ is the radar incidence angle. This hybrid SBAS–ISBAS framework enables robust deformation estimation in areas with variable coherence while maintaining consistency with the SBAS inversion scheme.

### 3.3. Atmospheric Correction

Tianjin is located in a coastal lowland environment, where atmospheric conditions are characterized by strong humidity variability and frequent convective activity. These factors can introduce significant phase delays in SAR observations and adversely affect deformation retrieval. To mitigate atmospheric artifacts, a spatiotemporal smoothing strategy was incorporated into the time-series inversion framework, exploiting redundancy among multiple interferograms acquired within close temporal intervals. The atmospheric phase screen (APS) was estimated using a data-driven averaging approach based on stable distributed scatterer (DS) pixels. Stable pixels were first identified using the amplitude dispersion index (ADI), with a threshold of 0.6 applied to select pixels exhibiting relatively stable amplitude behavior. These pixels are expected to contain limited deformation-related phase variation over short temporal intervals and are therefore suitable for atmospheric phase estimation. The selected stable pixels were used as control samples to interpolate the atmospheric phase over the entire scene [32].

For each acquisition date $t_{i}$, the atmospheric contribution $\alpha_{i}$ was estimated by averaging atmospheric residuals derived from interferograms formed with neighboring acquisitions within a finite temporal window $\left(t_{i-N},t_{i+N}\right)$. In practice, the window size *N* is constrained by the data availability and acquisition density of each SAR dataset. This finite-window formulation replaces the idealized infinite-sample assumption and allows practical implementation of APS estimation. The atmospheric phase for date $t_{i}$ can be expressed as

(3) $$\alpha_{i}=\frac{1}{2N}\sum_{j=1}^{N}∆\phi_{i\left(i-j\right)}-∆\phi_{i\left(i+j\right)}\;=\frac{1}{2N}\sum_{j=1}^{N}\left(N-j\right)\left[∆\phi_{\left(i-j\right)\left(i-j-1\right)}-∆\phi_{\left(i+j+1\right)\left(i+j\right)}\right]$$

where Δϕ represents the phase difference between interferogram pairs acquired at different times. In an ideal scenario, as *N* increases, the accuracy of the atmospheric phase estimate for each acquisition date improves.

To further suppress atmospheric noise and account for variability in interferogram quality, a normalized Atmospheric Noise Coefficient (ANC) was computed for each interferogram and used as a weighting factor during inversion. The factor of 10 serves as a scaling coefficient to enhance numerical stability. Interferograms exhibiting higher atmospheric variability were assigned lower weights, thereby reducing their influence on the final deformation estimates. The ANC is defined as

(4) $${ANC}_{i}=\left(10.0\right){\left(R_{max}\right)}^{-1}\sqrt{\frac{1}{M}\sum_{m=1}^{M}{\left(\alpha_{i}\left(X_{m}\right)-{\bar{\alpha }}_{i}\right)}^{2}}$$

where $R_{max}$ is the maximum coherence value and M is the number of interferograms used in the averaging process.

Following atmospheric correction, linear deformation rates and displacement time series were estimated for each DS point. Due to the presence of extensive low-coherence surfaces in Tianjin, including farmland, wetlands, and construction transition zones, the ISBAS strategy was employed to enhance spatial continuity by allowing intermittently coherent pixels to contribute to the time-series inversion.

Because the dominant deformation mechanism in the study area is land subsidence, which is primarily vertical in nature, the line of sight (LOS) deformation was projected into the vertical direction using the radar incidence angle:

(5) $$d_{vertical}=\frac{d_{LOS}}{cos\theta }$$

where $d_{vertical}$ is the quasi-vertical deformation, $d_{LOS}$ is the initially computed LOS deformation, and θ is the radar incidence angle. It should be noted that this projection represents an approximation assuming that horizontal motion is relatively small compared to vertical subsidence. This assumption is considered reasonable for long-term regional subsidence analysis in Tianjin and enables consistent comparison among multi-sensor SAR datasets with different viewing geometries.

### 3.4. Multi-Source SAR Data Fusion

Due to differences in acquisition time, orbital geometry, and signal-to-noise ratio among SAR sensors, direct concatenation of multi-source InSAR time-series can introduce artificial discontinuities. To analyze the long-term subsidence evolution over Tianjin from 2003 to 2024, we adopted a segmented calibration and global trend reconstruction strategy, which links temporally fragmented InSAR observations within a unified deformation framework rather than assuming continuous observations.

Firstly, the vertical deformation results derived from Envisat ASAR, ALOS PALSAR, and Sentinel-1 for their respective observation periods were resampled onto a common grid to ensure spatial consistency. For pixels covered by more than one dataset, deformation rates and epoch-wise differences during overlapping periods were extracted. Inter-sensor systematic offsets were then estimated using a least-squares approach and applied as baseline corrections to align the segmented time series.

After offset calibration, the deformation time series remain temporally fragmented due to data gaps between successive SAR missions. Instead of interpolating deformation during these gaps, we modeled the long-term cumulative deformation trend using a physically motivated continuous function. Considering that land subsidence in Tianjin is characterized by progressive development followed by deceleration and stabilization, a three-parameter S-shaped Logistic function, which has been widely applied in urban subsidence studies, was employed to describe the overall deformation evolution. An additional offset term was included to account for residual baseline differences among the segmented datasets [33]. Model parameters were estimated through an iterative least-squares optimization by minimizing residuals between corrected InSAR observations and the modeled deformation during periods with available data.

The logistic function used in this study is expressed as

(6) $$W\left(t\right)=\frac{W}{1+e^{-k\left(t-t_{0}\right)}}+b$$

where $W\left(t\right)$ represents the accumulated deformation at time t, W is the maximum possible deformation, and the parameters k and $t_{0}$ control the curve shape. To account for inter-sensor reference differences, an additive constant offset $b_{i}$ is introduced for each of the $W_{i}$ independent InSAR time series.

This fusion strategy does not treat individual datasets independently. Instead, it integrates bias calibration with global trend modeling to reconstruct a coherent long-term deformation trajectory from temporally segmented observations, providing a consistent basis for subsequent spatiotemporal analysis of ground subsidence evolution.

## 4. Results

### 4.1. Deformation Mapping for Whole Tianjin

**Figure 5:**
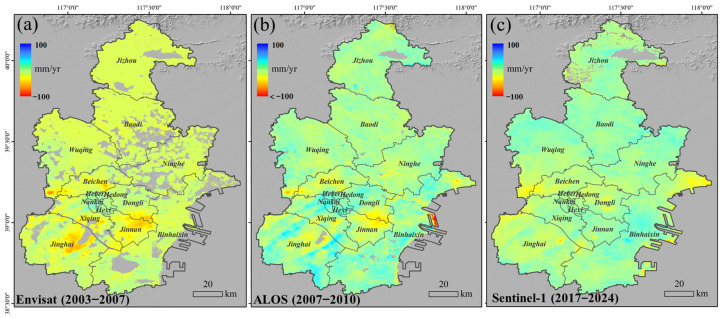
The vertical annual average velocity mapping of Tianjin. (**a**) Envisat ASAR from 2003 to 2007; (**b**) ALOS-PALSAR from 2007 to 2010; (**c**) S1 from 2017 to 2024.

Based on the processing techniques described in Section 3.2, the spatial distribution of the annual vertical deformation rates covering Tianjin from the three SAR datasets is shown in Figure 5. In Figure 5a, the annual deformation rate derived from Envisat ASAR is calculated for the period 2003–2007, which represents the non-overlapping early observation stage prior to the availability of ALOS data and is intended to highlight the initial spatial pattern of subsidence. Since 2003, pronounced subsidence funnels have developed in the urban core and surrounding districts, including Wuqing, Beichen, Jinnan, and Jinghai. The maximum subsidence rates in these regions exceeded 50 mm/yr, which is mainly attributed to long-term excessive groundwater extraction. From 2007 to 2010, subsidence rates in the southern parts of the city slowed a general deceleration, and the vertical deformation in the urban center was progressively reduced. In contrast, significant deformation persisted in coastal reclamation areas. Owing to limitations in data availability, the deformation results exhibit a temporal gap between 2011 and 2015. During the period from 2017 to 2024, subsidence within the main urban area was largely mitigated, while the dominant subsidence centers shifted toward the coastal reclamation zones of the Binhai New Area. In particular, areas such as Dongjiang Port and Fuzhuang remained affected by land subsidence, although the deformation rates showed an overall decreasing trend.

**Figure 6:**
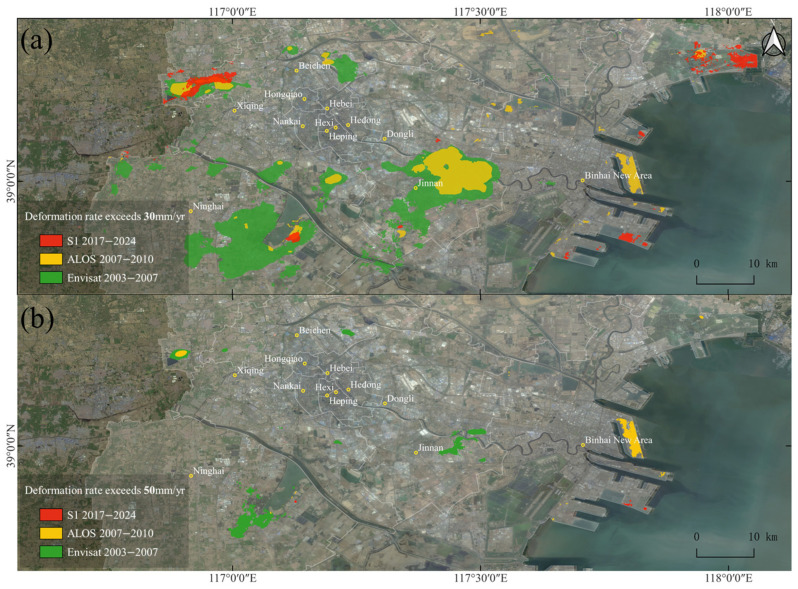
Subsidence classification statistics. (**a**) Distribution of vertical velocity rate exceeding 30 mm/yr. (**b**) Distribution of vertical velocity rate exceeding 50 mm/yr.

From the deformation rate results shown in Figure 5, it is evident that Tianjin’s subsidence follows a “banded distribution with multiple expanding centers” pattern. We further analyzed the spatial distribution of subsidence by calculating the areas where the deformation rates exceeded 30 mm/yr and 50 mm/yr, as shown in Figure 6. This analysis clearly highlights the coverage and subsidence trends of the four major subsidence centers, as well as newly emerging subsidence areas along the coast.

The Wuqing subsidence area is a continuous subsidence zone within the study region. It has expanded east and west along the Xiongjin Expressway, with the highest subsidence rate exceeding 30 mm/yr. During the first stage from 2003 to 2010, the maximum subsidence rate reached 58 mm/yr, affecting various schools and residential areas in Wangqingtuo Town. Currently, the subsidence rate in this area has been effectively mitigated, as seen in the S1 results. The Beichen subsidence area, centered around the North China Group, has shifted northwest towards Liu’anzhuang Primary School, with a maximum subsidence rate of 41 mm/yr. The subsidence here has been largely mitigated, and no significant deformation remains. The Jinnan subsidence center, which historically covered a large area, extends from Beifangzi to the surrounding regions, with the maximum subsidence rate reaching 57 mm/yr. Currently, subsidence continues in areas such as Zhonghai Park City and Nongjia Yi Strawberry Picking Park, with rates still exceeding 30 mm/yr. The Jinghai subsidence area, located around the Tuanbo Lake Reservoir, has experienced a maximum subsidence rate of 62 mm/yr. Present-day subsidence is mainly found in the newly developed area to the southeast of Tuanbo Lake Reservoir and in the reclamation areas within the lake, where subsidence rates were reduced to 54 mm/yr after 2017.

In addition to the four historical subsidence centers, new subsidence areas have emerged in the Binhai New Area, particularly around Dongjiang Port, Hairun Logistics Park, Tianjin Shipbuilding Manufacturing Company, and Dongdi Park. These areas are primarily coastal reclamation zones and are still undergoing the natural consolidation process of sediments, which has slowed the subsidence rates. Tianjin Tanggu Airport, a newly constructed large civil infrastructure project, has also caused some degree of surface deformation due to construction and operation activities. The area around Fuzhuang and Saijin Tuo Primary School, located on the northern side of Bohai Bay, has experienced the most severe subsidence, with a maximum subsidence rate of 43 mm/yr. This area is primarily agricultural and salt field land.

### 4.2. Consistency Analysis of Multiple-Source Data

Before merging the multi-source InSAR datasets, the consistency and reliability of the deformation results were comprehensively evaluated through a two-step validation strategy, including inter-sensor comparisons among different SAR datasets and external verification using independent ground-based leveling measurements. Overlapping regions among the Envisat ASAR, ALOS PALSAR, and Sentinel-1 datasets were selected for cross-validation to ensure the comparability of deformation rates derived from different sensors and acquisition geometries.

**Figure 7:**
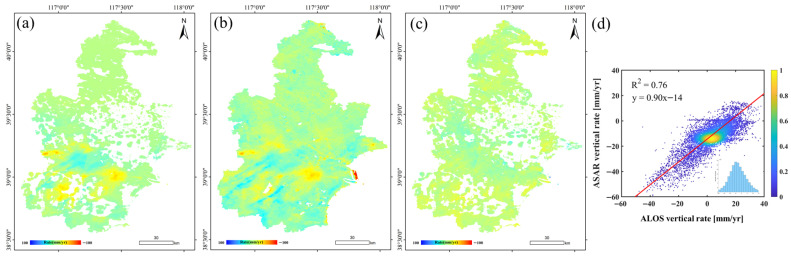
Cross-comparison of different sensors. (**a**) Envisat ASAR deformation rate during 2007–2010. (**b**) ALOS/PALSAR deformation rate during 2007–2010. (**c**,**d**) Spatial distribution and scatterplots of deformation rate residual between Envisat and ALOS.

Figure 7 presents the cross-comparison of deformation rates derived from different SAR sensors during their overlapping observation period from 2007 to 2010. Figure 7a,b shows the deformation rate maps obtained from Envisat ASAR and ALOS/PALSAR, respectively, which exhibit generally consistent spatial patterns. Figure 7c illustrates the spatial distribution of deformation rate residuals between the two datasets. Overall, the residuals are relatively evenly distributed across the study area; however, larger differences are observed in several major subsidence centers in southern Tianjin, where intense and spatially concentrated deformation occurs simultaneously in both datasets. These discrepancies are likely associated with sensor-dependent systematic differences. The quantitative comparison is further illustrated in the scatter plot shown in Figure 7d, where the correlation coefficient (R^2^) between Envisat and ALOS deformation rates reaches 0.76, indicating a strong level of consistency. The mean difference is −3.17 mm/yr, with a standard deviation of less than 5 mm/yr, suggesting that the two datasets are broadly comparable despite inherent sensor differences.

**Figure 8:**
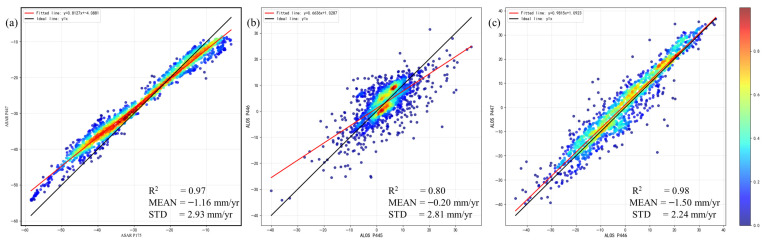
Cross-comparison of the same sensors. (**a**) Envisat P175 vs. P447; (**b**) ALOS P445 vs. P446; (**c**) ALOS P446 vs. P447.

Figure 8 shows the same-sensor cross-validation results based on different acquisition paths. Figure 8a–c presents the cross-comparisons between different orbital tracks of Envisat and ALOS. The correlation coefficients for these comparisons are all above 0.8, and both the mean differences and standard deviations are below 5 mm/yr. These results indicate a high level of internal consistency within each sensor dataset and support the reliability of the deformation estimates prior to multi-sensor time-series fusion.

**Figure 9:**
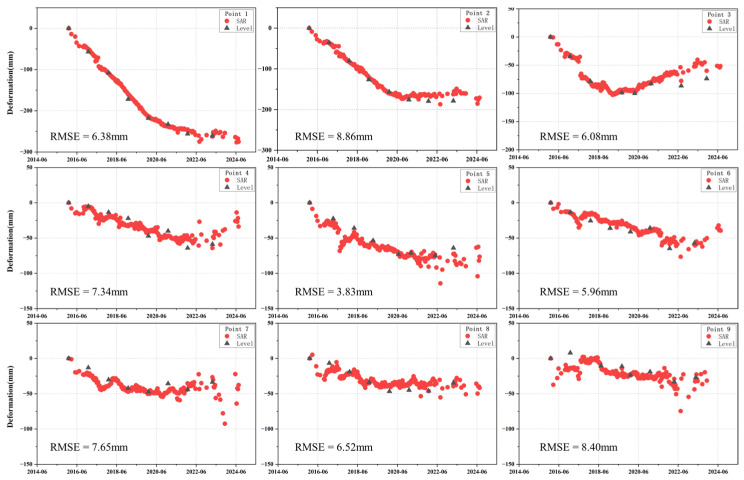
Cross-validation of the level temporal sequence with InSAR temporal sequence.

To further evaluate the reliability of the InSAR-derived deformation time series, independent ground-based leveling measurements from nine stations were used for external validation. Figure 9 shows the comparison between the Sentinel-1 InSAR time series and the corresponding leveling observations over their common observation period. The leveling data are provided as cumulative annual deformation. The first leveling epoch was used as the common reference for both datasets, and cumulative deformation during 2015–2023 was directly compared with the InSAR-derived vertical time series. The results indicate good agreement in both deformation magnitude and temporal trend. The root mean square error (RMSE) values for all stations are below 10 mm, with a maximum RMSE of 8.86 mm. This external validation confirms the reliability of the InSAR datasets prior to multi-source time-series fusion.

### 4.3. Fusion Results from Multiple Sensors

This section evaluates the performance of the multi-source fusion approach described in Section 3.4. By integrating Envisat ASAR, ALOS PALSAR, and Sentinel-1 datasets, a unified long-term deformation trajectory was reconstructed by linking temporally segmented InSAR observations from 2003 to 2024, enabling the overall subsidence evolution to be analyzed.

**Figure 10:**
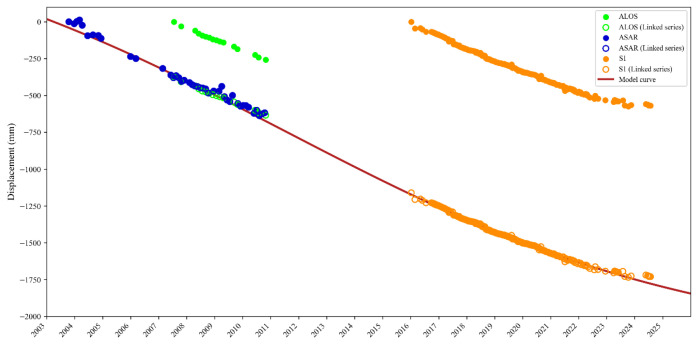
The segmented timing results of the settling point, the timing link results, and the Logistic model fitted curve.

Figure 10 illustrates the segmented deformation time series of a representative subsidence point, the temporal linkage among different datasets, and the fitted Logistic model curve. During the fusion process, the deformation time series were first aligned to a common temporal reference by fixing the offset of the earliest dataset. For clarity in visualizing the long-term deformation evolution, the fitted time series was subsequently shifted by a constant value so that the first available observation was set to zero. This normalization does not imply zero physical deformation at the initial epoch but serves only as a relative reference for comparing deformation evolution across different periods. All subsequent time series were referenced to this common baseline, allowing systematic offsets among different sensors to be effectively calibrated.

The reconstructed deformation trajectory exhibits good temporal consistency across different observation periods, and deformation rates derived from different sensors during overlapping intervals remain consistent. As shown in Figure 10, the available deformation observations mainly capture the linear deformation phase and the stabilization phase of subsidence, while the initial stabilization and acceleration stages of a complete S-shaped curve are not fully constrained by direct observations. Nevertheless, the fitted Logistic curve aligns well with the reconstructed deformation time series. Quantitative residual analysis between the corrected deformation time series and the fitted Logistic curve further supports the reliability of the fusion results. The mean residuals for the ALOS, Envisat ASAR, and Sentinel-1 datasets are 4.27 mm, 18.63 mm, and 1.65 mm, respectively.

These results indicate that the Logistic model provides a reasonable representation of the current subsidence evolution trend in Tianjin, particularly for areas dominated by linear and decelerating deformation phases. However, for grid units with relatively small deformation magnitudes, the applicability of the Logistic model still requires further validation, as also noted in previous studies [16].

## 5. Discussion

**Figure 11:**
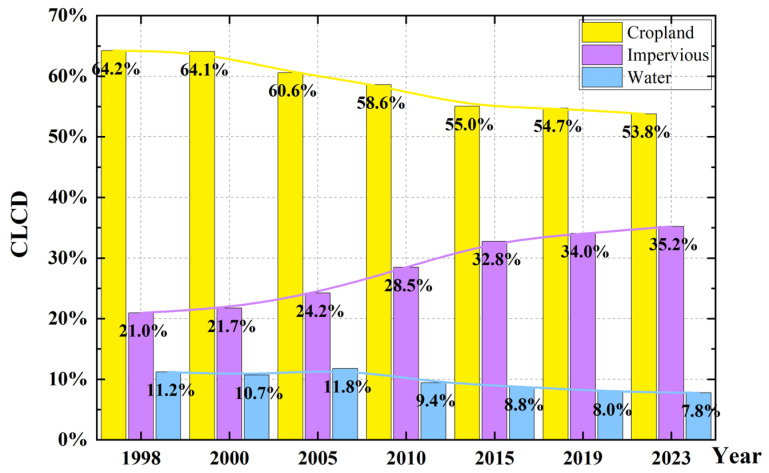
Statistical chart of the change in the proportion of CLCD in Tianjin from 1998 to 2023.

In Section 4.1, the spatiotemporal characteristics and regional differences in ground subsidence in Tianjin from 2003 to 2024 were revealed based on deformation rate results from multi-source InSAR data. To further investigate the causes of the changes in the spatiotemporal characteristics of subsidence in Tianjin, we used the 30 m resolution China Land Cover Data released by Yang Jie and Huang Xin from Wuhan University to statistically analyze the area changes and proportional distributions of three types of land-use in Tianjin: cropland, impervious surfaces, and water bodies [34]. The results shown in Figure 11. indicate that, based on land-use in 1998, by 2023 (25 years later), the area of cropland in Tianjin had decreased by more than 10%, the water body area had decreased by approximately 3%, and the impervious surface area had increased by 14.2%. This increase in impervious surfaces has already surpassed the combined decrease in cropland and water body areas. Thus, we discuss subsidence characteristics from two perspectives: urban expansion and coastal reclamation. These two factors are analyzed in the context of their contributions to the observed ground subsidence.

### 5.1. Coupling Relationship Between Urban Spatial Expansion and Ground Subsidence

The stage-wise variations in subsidence rates observed in central urban and expansion zones show a close spatiotemporal correspondence with urban construction activities and associated changes in surface loading. These regions are typically characterized by high construction density and frequent alterations in surface conditions. Large-scale residential and commercial developments may modify the stress state of near-surface soils, which can be reflected in progressive or slowly converging subsidence signals over time.

**Figure 12:**
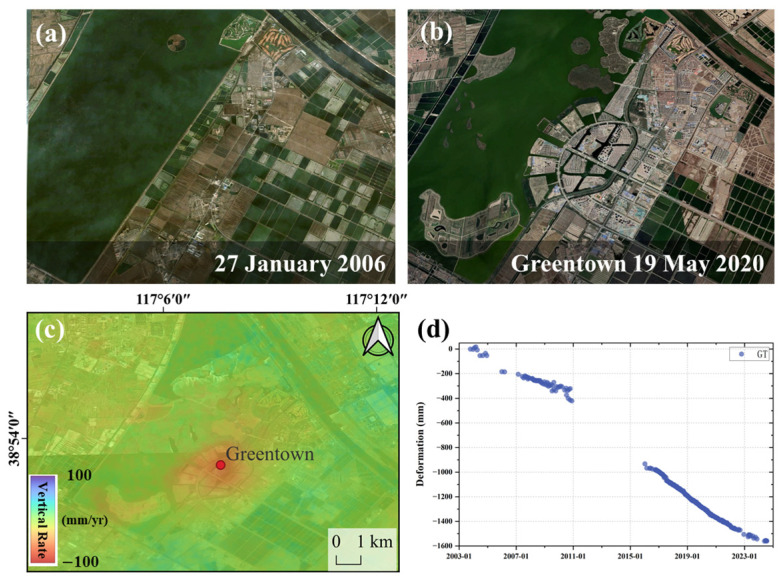
Greentown deformation along Tuanbo Lake, Jinghai. (**a**,**b**) Google images of Greentown. (**c**) S1 subsidence rate of Greentown. (**d**) Deformation time series of Greentown.

Taking the area around the Duliujiang River as an example, Google optical images show that significant construction and development began in 2006 on the east side of the Tuanbo Wetland Reservoir in Jinghai District, forming a new urban area with a high building density and concentrated residential areas. Figure 12 shows the temporal monitoring of the Green City, where the initial subsidence rate during the construction phase in 2007 was 30 mm/yr. From 2016 to 2023, the subsidence rate peaked at 40 mm/yr, after which the subsidence gradually stabilized, and the current subsidence rate remains at around 27 mm/yr. This temporal evolution is consistent with the cumulative effects of surface loading and subsequent foundation soil adjustment during urban expansion, although the exact contribution of individual processes cannot be quantitatively isolated.

**Figure 13:**
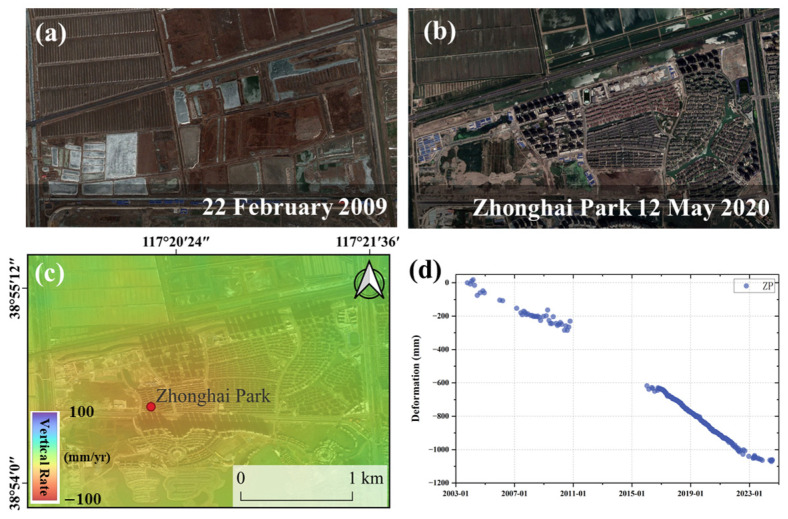
Zhonghai Park deformation in Jinghai. (**a**,**b**) Google images of Zhonghai Park. (**c**) S1 subsidence rate of Zhonghai Park. (**d**) Deformation time series of Zhonghai Park.

Another typical land-use conversion pattern during urban expansion is the transformation of farmland into construction land. This pattern is clearly demonstrated by the development of Zhonghai Park on the north side of Duliujiang River in the Tianjin Jinnan District. Figure 13d shows that before 2009, the area to the north of Tianjia Lake was mainly farmland and excavation pits, with a subsidence rate of 14 mm/yr, indicating a relatively stable state. During the concentrated construction phase of the residential buildings, the subsidence rate significantly increased to about 38 mm/yr. By July 2022, the subsidence rate began to level off, decreasing to around 5 mm/yr. Compared with the wetland-to-construction land transition, the observed subsidence convergence in the farmland-to-construction land case occurred earlier, suggesting faster soil consolidation. This interpretation is based on observed deformation characteristics.

Overall, the gradual conversion of farmland, wetlands, and other landforms into construction land is closely associated with the stage wise evolution of subsidence. The observed deformation patterns suggest that cumulative surface loading and foundation soil consolidation are important contributing factors in urban expansion areas, while acknowledging that explicit causal relationships cannot be fully established without independent geotechnical or hydrological constraints.

### 5.2. Spatiotemporal Heterogeneity of Subsidence in Coastal Anthropogenic Activities

**Figure 14:**
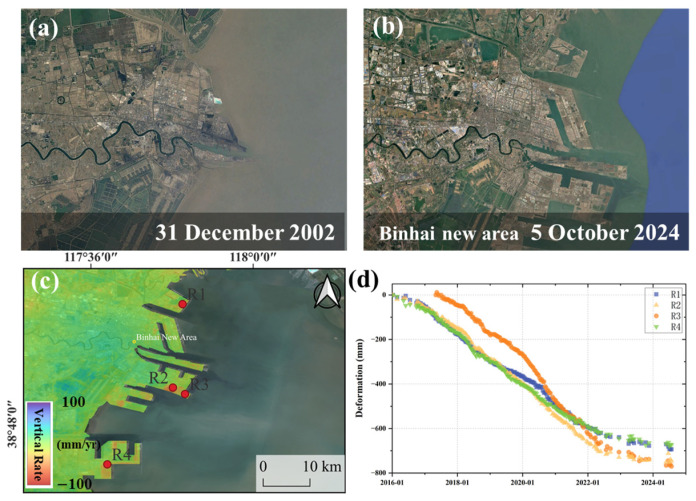
Land reclamation subsidence in Binhai New Area. (**a**,**b**) Google images in different times. (**c**) The deformation rate of Land reclamation in Binhai New Area. (**d**) Time series of four feature points in land reclamation area.

Beyond urban expansion, coastal anthropogenic activities, particularly large-scale reclamation and aquaculture development, are closely associated with subsidence and exhibit pronounced spatiotemporal heterogeneity. In recent years, extensive reclamation activities have been carried out in the coastal areas of Tianjin Binhai New Area. The newly reclaimed land primarily consists of soft clay and incompletely consolidated alluvial and sedimentary deposits, which are generally susceptible to post-construction deformation due to their physical and mechanical properties. The InSAR monitoring results show significant uneven subsidence in the region (Figure 5), with the subsidence evolution exhibiting a rapid initial subsidence and followed by a slowdown pattern (Figure 14). This evolution is consistent with the typical consolidation behavior of soft soils. The time-series results indicate that deformation convergence in four representative reclamation areas occurred around April 2022. However, as reclamation projects in these areas have not yet been fully completed and land-use planning has not been fully implemented, the apparent stabilization should be interpreted cautiously. Continued long-term monitoring is therefore required. It is also noteworthy that, despite partial convergence, localized zones still exhibit subsidence rates exceeding 15 mm/yr, which may pose potential risks to the long-term stability of port facilities, roads, and stockpiling infrastructure.

**Figure 15:**
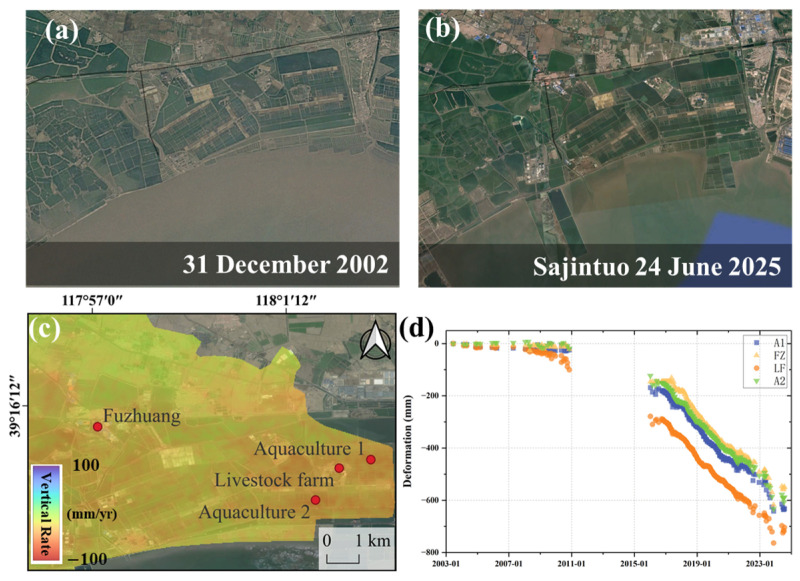
Subsidence of the Sajintuo Livestock and Aquaculture Areas in Binhai New Area. (**a**,**b**) Google images of Fuzhuang at different time periods. (**c**) Deformation rate and feature point locations of the livestock and aquaculture farms. (**d**) Time series of the feature points.

Subsidence patterns associated with coastal aquaculture and livestock farming areas exhibit additional complexity. Prior to construction, deformation in these regions remained relatively stable. Following the initiation of aquaculture operations, sustained high subsidence rates of approximately 40–45 mm/yr were observed (Figure 15). Time-series analysis shows short-term stagnation of subsidence at Fuzhuang after January 2019, while subsidence rates in areas A1, A2, and the livestock farming zones decreased between 2020 and 2021. These changes may be associated with construction suspension and reduced operational activity during the COVID-19 pandemic. In aquaculture and livestock farming areas, groundwater extraction is typically required for salinity control and daily water use. Subsidence related to groundwater extraction often exhibits a delayed response, which may partly explain the observed lag in subsidence deceleration.

Overall, uneven subsidence in the coastal reclamation areas of the Tianjin Binhai New Area is closely linked in space and time to soil consolidation characteristics and anthropogenic engineering activities, including reclamation, aquaculture facility construction, and groundwater use. The observed subsidence phases show strong correspondence with construction timelines, external disturbances, and variations in activity intensity. Localized ongoing subsidence highlights the need for targeted mitigation strategies and sustained long-term monitoring, ideally integrated with soil mechanics and engineering activity assessments.

In a broader global context, the magnitude and spatiotemporal evolution of land subsidence observed in Tianjin are consistent with patterns reported in other rapidly urbanizing and coastal or deltaic regions worldwide. Previous studies have documented subsidence rates on the order of several tens of millimeters per year in major coastal megacities such as Shanghai, Jakarta, and the Tokyo Bay area, where intensive groundwater exploitation, large-scale land reclamation, and rapid urban expansion are dominant driving factors. Similar to these regions, Tianjin exhibits a clear transition in subsidence mechanisms, from groundwater-induced consolidation in inland urban districts to engineering- and reclamation-related deformation in coastal zones [2,3,35]. This consistency suggests that the subsidence processes identified in Tianjin reflect common challenges faced by coastal megacities undergoing accelerated land-use transformation, supporting the broader applicability of the monitoring framework presented in this study.

## 6. Conclusions

This study establishes a long-term land subsidence analysis framework for Tianjin by integrating multi-source SAR observations from Envisat ASAR, ALOS PALSAR, and Sentinel-1 using SBAS-InSAR processing, atmospheric correction, and logistic model-based time-series fusion. Although continuous SAR observations are not available throughout the entire study period, the proposed fusion strategy enables the reconstruction of the long-term deformation trend and its staged evolution characteristics from 2003 to 2024.

The results indicate that, during the period from 2003 to 2010, subsidence bowls were mainly concentrated in Wuqing, Beichen, Jinnan, and Jinghai, with maximum subsidence rates reaching approximately 50 mm/yr. This stage was primarily controlled by long-term excessive groundwater extraction. After 2017, following the implementation of groundwater exploitation restrictions and ecological water replenishment policies, subsidence in these inland urban areas was effectively mitigated. In contrast, the dominant subsidence centers gradually shifted toward coastal reclamation zones in the Binhai New Area, particularly Dongjiang Port and Fuzhuang, where land reclamation and urban expansion became the main driving factors. In these areas, soil consolidation induced by newly imposed surface loading played a critical role in controlling the staged subsidence behavior.

The multi-source SAR fusion strategy adopted in this study effectively addressed the temporal discontinuities and systematic differences among sensors, enabling the reconstruction of the long-term deformation evolution trend of Tianjin over two decades. This integrated framework provides valuable insights into the long-term response of urban and coastal environments to groundwater regulation, land-use transformation, and large-scale engineering activities.

Despite these advances, several limitations should be acknowledged. Temporal gaps remain unavoidable due to the non-overlapping acquisition periods of different SAR missions, and independent validation of the reconstructed long-term fused time series is constrained by the lack of continuous in situ observations covering the entire study period. In addition, the analysis of multiple driving mechanisms was primarily based on spatiotemporal correlations with land-use changes and policy interventions, while explicit geo-mechanical and hydrogeological modeling was not incorporated. Future research should focus on integrating physical models with multi-source InSAR observations, strengthening long-term validation using continuous ground-based measurements, and extending the current framework toward dynamic risk assessment and early-warning systems for subsidence-prone coastal cities.

## Figures and Tables

**Table 1:** Basic parameters of three satellite SAR datasets.

SAR Sensor	Band (Wave)/cm	Polarization/ Orbital Direction	Observation Mode	Resolution (Rg × Az)/m	Track	Frame	Acquisition Time Span	No. of Images
Envisat ASAR	C (5.6)	VV/Descending	Image Mode (IM)	30 × 30	175	2817	15 June 2003–26 September 2010	30
					447	2799	17 October 2003–15 October 2010	39
						2817	17 October 2003–15 October 2010	47
						2835	26 December 2003–15 October 2010	47
ALOS/PALSAR	L (23.6)	HH/Ascending	Fine-Beam Single polarization (FBS) Fine-Beam Double polarization (FBD)	20 × 10 10 × 20	445	770	29 January 2007–9 November 2010	19
						780	29 January 2007–9 November 2010	19
						790	29 January 2007–9 November 2010	13
					446	760	31 December 2006–26 November 2010	23
						770	31 December 2006–26 November 2010	23
						780	31 December 2006–26 November 2010	23
						790	31 December 2006–26 November 2010	18
					447	760	17 January 2007–31 December 2010	19
						770	17 January 2007–31 December 2010	19
						780	17 January 2007–28 October 2010	18
Sentinel-1	C (5.6)	VV/Ascending	Interferometric Wide-swath (IW)	5 × 20	69	124	9 January 2016–25 July 2024	190
						129	3 May 2017–25 July 2024	161

## Data Availability

Data will be made available on request.
